# A good start of immunotherapy in esophageal cancer

**DOI:** 10.1002/cam4.2336

**Published:** 2019-06-23

**Authors:** Qian Zhao, Jinming Yu, Xue Meng

**Affiliations:** ^1^ Cheeloo College of Medicine ShanDong University Jinan China; ^2^ Department of Radiation Oncology Shandong Cancer Hospital affiliated to Shandong University, Shandong Academy of Medical Sciences Jinan China

**Keywords:** adoptive T‐cell transfer, biomarker immune checkpoint inhibitor, esophageal cancer, immunotherapy, oncolytic virus, peptide vaccine

## Abstract

Considering the benefits of immunotherapy in advanced melanoma, non–small cell lung cancer, renal cell carcinoma, bladder cancers, and refractory Hodgkin lymphoma, we begin to consider whether immunotherapy is effective for esophageal cancer, which is extremely malignant and has a poor prognosis. There are a large number of clinical trials to study the application of immunotherapy such as immune checkpoint inhibitors, peptide vaccine, adoptive T cell transfer and oncolytic virus in esophageal cancer. Some already have preliminary results and show the advantages of immunotherapy in esophageal cancer, while others are still in progress. This review aims to introduce the feasibility and current status of immunotherapy in esophageal cancer.

## 1 BACKGROUND

Esophageal cancer (EC) is the eighth most common cause of cancer‐related death worldwide and seriously threatens human health.^1^ At present, the treatment of esophageal cancer in the clinic mainly includes surgery, chemotherapy, radiotherapy, targeted therapy and a combination of them. The use of neoadjuvant chemoradiotherapy and targeted therapy has improved overall survival. However, because approximately half of the patients have distant metastases when esophageal cancer is clinically diagnosed, surgery is no longer applicable.^2^ Chemotherapy based on 5‐fluorouracil，platinum agents，and taxanes combined with radiotherapy has become the standard treatment for advanced esophageal cancer. However, it has been shown that esophageal squamous cell carcinoma (ESCC) and esophageal adenocarcinoma (EAC) are inherently resistant to systemic therapy due to histology, molecular and etiological heterogeneity, with limited responses seen after first‐line therapy.^3^ The application of targeted drugs is very limited in esophageal cancer，and is only administered in EAC targeting HER2 or vascular endothelial growth factor^4^, ^5^, ^6^，and there is no current evidence showing that targeted therapy has an obvious benefit in ESCC. Although the traditional treatments have been improved, the 5‐year global survival is still poor at 30%‐40%.^7^ We urgently need new treatments to improve the prognosis of this disease. The emergency of immunotherapy has gradually attracted oncologists’ attention, and its application in melanoma, lung cancer, and kidney cancer has revolutionized their management.^8^ We expect that immunotherapy is also applicable in the treatment of esophageal cancer. However, there are still some problems in the application of immunotherapy to esophageal cancer.

This review focuses on what is the biological basis of esophageal cancer for immunotherapy, how to screen the patients who can benefit from immunotherapy, and whether the toxic side effects of immunotherapy are manageable.

## 2 THE BIOLOGICAL BASIS OF IMMUNOTHERAPY IN ESOPHAGEAL CANCER

Under normal physiological conditions, the body's immune response is regulated by the costimulatory and inhibitory signals. When the immune system is activated by the stimulation signals of pathogens, it can accurately identify specific antigens and eliminate them. Normal tissues can prevent the damage of the immune system by expressing immune checkpoints which we call self‐tolerance. After a long period of debate about whether the immune system can specifically recognize and kill tumor cells, much evidence shows that immune cells do play a significant role in the control of tumor cells. For example, we observed that people who are immunocompromised are prone to cancer, which is also confirmed in animal models. The accumulation of immune cells can be observed at the tumor site which is usually associated with prognosis. As technology advances, immune responses can be detected directly in patients.^9^, ^10^, ^11^ Under ideal conditions, the antigens on the surface of the tumor cells will induce inflammation and be recognized and phagocytized by antigen presenting cells (APC), especially dendritic cells (DC), and then presented to T lymphocytes and B lymphocytes, triggering an adaptive response.^12^ However，despite the well‐established immune mechanism, malignant tumors still occur.

Tumor cells have multiple strategies to resist immune surveillance which we call immune evasion. The most well‐known and most studied are the immune checkpoints which are expressed on the surface of tumor cells, such as programmed cell death 1 ligand 1 (PD‐L1) and cytotoxic T lymphocyte‐associated antigen‐4 (CTLA‐4), which can bind to receptors on immune cells and act as an inhibitory signal to suppress immune cells function.^13^ Recently, several studies have shown that PD‐L1 can also be expressed on extracellular vesicles (EVs) secreted by cancer cells,^14^, ^15^ and the expression level is related to tumor progression. Exosomes are one special form of AEs, which could carry PD‐L1 to drain lymph node and then suppress T‐cell function. However, immune checkpoint inhibitor has no effect on exosomes.^16^ Maybe in the future, this will be a new target for tumor immunotherapy. Additionally, tumor cells or tumor‐associated macrophages (TAMs) could also secrete CCL17 and CCL22 which are able to recruit CCR4^+^ regulatory T cells (Treg). Treg cells have different roles in normal tissues and tumor tissues. In tumor tissues, Treg cells can finally result in the occurrence and progression of tumor cells. Myeloid‐derived suppressor cells (MDSCs) can be stimulated by inflammation and tumor‐derived factors which directly inhibit the expression of CD8^+^T cells. Some stromal cells in the tumor microenvironment inhibit the body's immune system function, leading to tumor progression and metastasis.^17^ Based on the aforementioned mechanisms of tumor immune evasion we currently have two ways to fight tumor cells with the autoimmune system, agonists of costimulatory receptors and antagonists of inhibitory signals.^13^ They both enhance the specific antitumor effect of the immune system.

The main risks for ESCC are smoking and alcohol abuse which may induce gene mutations which can be easily recognized by the immune system. The well‐established association of precursor chronic inflammatory lesions and high gene mutation rates with approximately 3000‐300 000 mutations per tumor gives the rationale for developing immunotherapy in esophageal cancer.^18^ Currently, pembrolizumab was recommended for the treatment of esophageal and esophagogastric junction (EGJ) adenocarcinoma with high microsatellite instability or deficient mismatch repair or PD‐L1‐positive. A large number of clinical trials to study the application of immunotherapy such as immune checkpoint inhibitors, peptide vaccine, adoptive T‐cell transfer, and oncolytic virus are currently underway.

## 3 SEVERAL CLINICAL TRIALS OF IMMUNOTHERAPY FOR ESOPHAGEAL CANCER

### 3.1 Immune checkpoint inhibitor

Pembrolizumab，an inhibitor of PD‐1，was the first immune checkpoint inhibitor approved in 2014 by the FDA based on the phase Ib KEYNOTE‐001 for the treatment of advanced or unresectable melanoma,^19^, ^20^ more recently for recurrent or metastatic squamous cell carcinoma of the head and neck, for advanced non‐small cell lung cancer expressing PD‐L1 as second‐line therapy and also for chemotherapy‐refractory PD‐L1‐positive gastric/GEJ cancer.^21^, ^22^, ^23^, ^24^, ^25^ KEYNOTE‐028, a multicohort, phase IB study, was designed to evaluate the safety and overall response rate of pembrolizumab in PD‐L1 positive advanced solid tumors. Twenty‐three patients with PD‐L1‐positive, advanced and metastatic esophageal cancer were enrolled which consisted of 17 ESCCs，five EACs and one mucoepidermoid carcinoma. Patients received 10 mg/kg pembrolizumab intravenously every 2 weeks for up to 2 years or until disease progression intolerable toxicity. There were no unexpected treatment‐related adverse events and more than half of the patients had tumor shrinkage from baseline. Objective response rate (ORR) was 30% (95% CI,13%‐53%) which consisted of 5 ESCCs and 2 EACs and they were all confirmed partial response (PR). Two patients were confirmed with stable disease (SD). There were no unexpected treatment‐related adverse events and more than half of the patients had tumor shrinkage from baseline. Because of the small number of adenocarcinoma patients, it is impossible to draw conclusions about progression‐free survival (PFS) or overall survival (OS) through histological subtypes. Of interest, this trial suggests that gene expression profiling may associate with the efficiency of pembrolizumab.^26^ KEYNOTE‐180, an open‐label, phase 2, international study, was designed to evaluate the safety and efficacy of pembrolizumab. 121 patients with advanced, metastatic esophageal cancer were enrolled and they received 200mg pembrolizumab every 3 weeks for up to 2 years or until unacceptable toxic effects, disease progression. The results of the experiment were very satisfactory. The ORR was 9.9% (95% CI, 5.2%‐16.7%) and they all had a partial response. Median OS was 5.8 months and the 6‐month and 12‐month OS rate were 49% and 28%, respectively. Forty three had tumor reduction from the baseline. This result is much more encouraging than the results of previous traditional second‐line treatment setting.^27^ These data bring benefit to patients who had progressive disease (PD) after two or more lines of therapy. The authors also found that pembrolizumab is also effective in patients with negative PD‐L1 expression and its effectiveness is not associated with histologic characteristics.^28^ KEYNOTE‐181 study，a phase III clinical trial，was aimed to evaluate pembrolizumab versus chemotherapy as second‐line therapy for advanced esophageal cancer. In this study, pembrolizumab shows a clinically meaningful improvement in OS,with a better safety profile, which may have implication for pembrolizumab as second‐line standard therapy for EC with PD‐L1 positive (combined positive score ≥10).^29^ Another phase III KEYNOTE‐590 study is investigating pembrolizumab combined chemotherapy versus placebo combined chemotherapy as first‐line treatment for advanced EC.

Nivolumab, a fully human IgG4 monoclonal antibody targeting PD‐1, has a high affinity for PD‐1 which can inhibit the binding of PD‐L1/PD‐L2 to PD‐1.^30^ Nivolumab has been approved by the FDA to treat metastatic melanoma, non‐small cell lung cancer, and renal cell carcinoma.^31^ ATTRACTION‐2, a randomized, double‐blind, phase 3 trial, was designed to evaluate the efficacy and safety of nivolumab in patients with chemotherapy‐refractory gastric and gastroesophageal junction (GEJ) cancers in Japan. This study demonstrated that nivolumab has statistically and clinically significant benefits for patients who have received two or more lines of chemotherapy and have not been selected for PD‐L1 expression.^32^ In Japan, another study also reported a preliminary result of the safety and activity of nivolumab in patients with esophageal cancer. Sixty four patients with treatment‐refractory esophageal cancer were enrolled, all diagnosed with squamous cell carcinoma. Patients received 3 mg/kg of nivolumab intravenously once every 2 weeks in 6‐week cycles and the primary outcome was centrally assessed ORR. Eleven (17%, 95% CI 10‐28) had an ORR by central assessment and 14 (22%, 14‐33) by investigator assessment. The median overall survival (mOS) was 10.8 months (95% CI 7.4‐13.3) and the median progression‐free survival (mPFS) was 2.9 months (95% CI 1.9‐5.6). No deaths were related to treatment. Nivolumab has shown promising activity and manageable safety. In this study, the authors challenge the applicability of RECIST and WHO criteria to evaluate the tumor response of immunotherapy because immune‐related response requires confirmation after disease progression.^33^ It has indeed been confirmed that there would be increased tumor burden or appearance of new lesions after immunotherapy. Establishing the most appropriate criteria for assessing the activity of immunological checkpoint inhibitors is necessary.^34^, ^35^ Since the non‐overlapping mechanism of anti‐PD1 and anti‐CTLA4 antibodies, and the clinical response of the combination of these two checkpoint inhibitors showed improvement,^36^ CheckMate 032 study first proposed a combination treatment of nivolumab and ipilimumab in esophagogastric cancer. In this study, the clinical response of nivolumab monotherapy was consistent with the ATTRACTION‐2 study and nivolumab plus ipilimumab was superior to nivolumab monotherapy. The effectiveness of the inhibitor was not related to the expression of PD‐L1. We need further studies to determine an optimal approach of when and how to combine nivolumab and ipilimumab. Several phase III studies, NCT02743494, CheckMate 648 and CheckMate 649 trial are currently under way.^37^, ^38^

### 3.2 Anti‐CTLA‐4

CTLA‐4 is a transmembrane receptor on T cells. When it binds to CD80 or CD86 the immune system is downregulated.^39^ Currently, antibodies targeting CTLA4 are widely used in many forms of tumors.^40^ Tremelimumab, a humanized monoclonal antibody against CTLA4, was tested in metastatic melanoma as the second‐line setting. Although its efficacy is not ideal, its combination with PD‐1 inhibitors has indeed attracted attention.^8^, ^41^ Ipilimumab，another monoclonal antibody, activates the immune system by targeting CTLA4. The combination of nivolumab and ipilimumab has demonstrated synergy in preclinical models. CheckMate‐032 study has demonstrated the safety and efficacy of nivolumab plus ipilimumab in patients with advanced esophageal cancer and confirmed that nivolumab plus ipilimumab was superior to nivolumab monotherapy. Many clinical trials of the combination of CTLA4 and PD‐1 inhibition are in progress. However, the side effects of blockade of CTLA4 are more common and more serious than PD‐1/PD‐L1; development of new strategies to reduce serious adverse events is underway.^40^

### 3.3 Adoptive T‐cell transfer

Another well‐known immunotherapy is adoptive T‐cell therapy which is a form of passive immunization. Activated T cells are usually collected from cancer tissue which is known as infiltrating lymphocytes and peripheral blood vessels. The isolated T cells are stimulated by IL‐2 in vitro and then infused back to the patients. Another type includes genetically engineered T cells, translocating chimeric antigen receptor (CAR‐T cells) or transducing the antigen‐specific T cell receptor (TCR) into T cells (TCR‐T cells). The purpose is to improve tumor‐specific immunity.^34^, ^42^ Many trials have shown that the persistence of adoptive T cells was related to the regression of tumors.^43^ Therefore, to enhance the persistence of autologous cells in humans has become a major obstacle to the application of effective cell transfer therapy. Preparative lymphodepletion combined with chemotherapy or chemoradiotherapy was confirmed to increase the existence time of adoptive T cells.^44^ The first clinical trial of adoptive cell therapy (ACT) for the patients with advanced or recurrent EC was arranged in 2000. The patients received the 0.8 × 10^9 ^activated lymphocytes every 2 weeks. The lymphocytes were administrated into primary tumors, metastatic lymph nodes, pleural spaces or ascitic regions. This study reported that four of 11 patients had a significant tumor regression and this treatment profile was safe.^45^ The first‐in‐man clinical trial of TCR T‐cells transfer in recurrent melanoma‐associated antigen 4(MAGE‐A4)‐expressing ESCC did not include preparative lymphodepletion. Although transferred T cells persisted for a long time and the tumor‐specific reaction was maintained, seven patients showed tumor progression after 2 months. This discordance between T‐cell persistence and tumor regression indicates that preparative lymphodepletion enhances antitumor responses via multiple mechanisms. Three patients with minimal lesion survived more than 27 months after treatment which suggests that TCR T‐cells transfer might be beneficial for minimal tumors.^46^

### 3.4 Peptide vaccine

Since several immunogenic cancer antigens (ICA) were identified on ESCC cells, researches of therapeutic cancer vaccine have been conducted globally. Cancer vaccines are designed to effectively induce cancer antigen‐specific cytotoxic T lymphocytes and enhance immune response.^37^, ^47^ K.Mimura et al performed phase I and II clinical trials of cancer vaccine using three human leukocyte antigen‐A24 (HLA‐A24)‐binding peptides from TTK protein kinase (TTK), lymphocyte antigen‐6 complex locus K (LY6K), and insulin‐like growth factor‐II mRNA binding protein‐3 (IMP3). The phase II clinical trial firstly showed a promising result of therapeutic cancer vaccine with multiple peptides.^48^ Another target for cancer vaccine is New York esophageal squamous cell carcinoma‐1(NY‐ESO‐1). Kawabata R, et al reported the first clinical trial of cholesterol‐bearing hydrophobized pullulan (CHP)‐NY‐ESO‐1 vaccination for patients with esophageal cancer. The study showed that a dose of 100 μg formulation of CHP‐NY‐ESO‐ 1 protein resulted in the increase of NY‐ESO‐1 antibody responses.^49^ In one study, patients with advanced ESCC who underwent neoadjuvant therapy followed by curative resection received peptide vaccine. Patients who received cancer vaccine tend to show better 5‐year esophageal cancer‐specific survival than those not receiving cancer vaccine.^50^ Clinical trials of individualized peptide vaccines that target individual neoantigens are now in process in different solid tumors.^51^

### 3.5 Oncolytic virus

Many experts believe that oncolytic virus therapy is perhaps another breakthrough in immunotherapy after the success of ICI.^52^, ^53^ Oncolytic viruses selectively replicate in cancer cells and then induce tumor cells lysis. In October 2015, talimogene laherparepvec (or T‐Vec), the first oncolytic virus showed clinical benefits and was approved in advanced melanoma patients.^54^ Recently, one phase I/II study was designed to further evaluate the efficacy of a novel telomerase‐specific oncolytic virus, telomelysin (OBP‐301), in combination with locoregional radiotherapy in elderly ESCC patients. This study demonstrated the efficiency and toleration of OBP‐301.^55^ Some other clinical trials of various oncolytic viruses in EC patients are ongoing.

## 4 HOW TO SCREEN THE PATIENTS WHO CAN BENEFIT FROM IMMUNOTHERAPY?

In the above experiments, we found that not all patients could benefit from immunotherapy. Among the unscreened and immunotherapy‐treated patients, only a subset of patients achieved significant improvement in overall survival and progression‐free survival. A number of clinical trials have shown that the therapeutic effect of PD‐1/PD‐L1 immunological checkpoint inhibitors correlates with PD‐L1 expression levels in the patient's tumor microenvironment.^56^ In addition, considering the many side effects and the high price of immunotherapy, it is especially important to find reliable checkpoints before performing immunotherapy in esophageal cancer. The most widely used biomarkers currently known are pd1/pdl1, tumor‐infiltrating lymphocytes (TIL), microstatellite instability (MSI), tumor mutation burden (TMB) and the like.

PD‐L1 also known as cluster of differentiation 274 (CD274) or B7 homolog 1 is one of the transmembrane proteins. PD‐L1，a ligand for PD‐1，which is expressed on dendritic cells and tumor cells， bind to PD‐1 which is expressed on T cells. When they are combined, the activation of antigen‐driven T cells can be inhibited. This is also one of the mechanisms of tumor immune evasion. Anti‐PD‐L1 inhibitors could bind to PD‐L1 and then inhibit the combination of PD‐1 on T cells and PD‐L1 on tumor cells，which allows immune cells to recognize and kill tumor cells. Immune checkpoint inhibitors have been successfully used in the treatment of advanced melanoma, non–small cell lung cancer, renal cell carcinoma, bladder cancers, and refractory Hodgkin lymphoma. We hope that immune checkpoint inhibitors can also perform well in the treatment of esophageal cancer. PD‐L1 expression rate as reported in ESCC ranges from 41.9% to 84.5%.^6^ PD‐L1 positivity in tumor cells and tumor‐infiltrating immune cells has shown prognostic value.^57^ It has been reported that the expression of PD‐L1 in tumor cells and tumor‐infiltrating immune cells is significantly associated with good overall survival, however, some reports are just the opposite.^6^, ^8^, ^37^, ^42^, ^58^, ^59^ The better the tumor differentiation, the negative lymph node metastasis and the early stage tumors have a higher expression rate of PD‐L1. But some reports are just the opposite. One meta‐analysis included 3306 patients with EC yielded summary statistics indicating that PD‐L1 overexpression has an unfavorable impact on OS, with the pooled HR of 1.42 (95% CI: 1.09‐1.86), but not on disease‐free survival (DFS) (HR  =  1.08, 95% CI: 0.76‐1.53). However, two studies included in this analysis showed PD‐L1 expression to be a favorable prognostic factor for OS in EC. The authors suspect that different heterogeneous baseline characteristics, diverse primary antibodies used，the definitions of positive staining applied and the cut‐off values adopted could lead to the discordant results.^60^

Cytotoxic T lymphocyte‐associated antigen‐4(CTLA‐4) is a transmembrane receptor on T cells, which shares the B7 ligand with CD28, while CTLA‐4 binds to B7 to induce T cell anergy and participate in the negative regulation of immune response. Positive rate expression of CTLA4 is associated with overall survival, and the higher the expression density, the shorter the survival.^61^ However, because only one study reported the relationship between CTLA‐4 expression and overall survival of ESCC, we expect more experiments to help figure out the relationship between them.

The mutations or epigenetic inactivation of the mismatch repair (MMR) genes MLH1, MSH2, MSH6, and PMS2 could cause microsatellite instability (MSI),^62^ which means that the microsatellite sequences of DNA cannot be repaired，resulting in a replication error. Tumor mutation burden is related to MSI and they both have reliable predictive and prognostic value.^63^ High TMB means that more new antigens are exposed to the body's immune system, which can better activate the immune system and make ICIs treatment more effective, which has been verified in metastatic colorectal cancer.^64^ It has been reported that esophageal cancer is a tumor with a high mutation load.^18^ Thus, we could expect ICIs to be efficient in the treatment of esophageal cancer. However, in esophageal cancer, MSI rarely happened, and it may be not effective for the predictability of ICIs.

The tumor microenvironment(TME) includes fibroblasts, bone marrow‐derived inflammatory cells, immune cells, lymphocytes, etc^65^ The microenvironment of each tumor has its own inflammatory characteristics. Tumor‐infiltrating lymphocytes (TILs) in TME have shown a good prognosis associated with breast, melanoma and colorectal cancer.^8^ Galon, J est. proposed a concept of immunoscore which not only refers to the tumor's TNM staging but also includes TILs.^66^ It is a scoring system which is used to describe the density of CD3+ and CD8+ T cells and their invasive margin.^67^ One study investigated the relationship between TILs and PD‐L1 expression and the prognosis of esophageal cancer. This study collected 53 banked tissue specimens and all the tumors were adenocarcinomas. They found high densities of CD8^+^TILs and CD3^+^TILs had no effect on survival and recurrence. Other experiments have confirmed that cytotoxic T cells in gastrointestinal malignancies are not the main cell types of TILs. Establishing a new immunoscore in esophageal cancer remains a challenge.^58^ Another marker in TME that effectively predicts prognosis is the neutrophil‐lymphocyte ratio (NLR). The NLR has been studied as a general predictor of therapeutic effect.^21^ Lymphocyte counts represent the effect of tumor immunity, and neutrophils play a role in negative chronic inflammation, so low NLR seems to be more effective for ICIs. However, it remains controversial. Recently a study in Japan examined neutrophils, %Tim3, %OX40, and so forth after the first cycle treatment of Nivolumab in ESCC. Twenty patients with esophageal cancer were enrolled in this clinical trial. They concluded that the increase of Tim‐3 in T cells may serve as a marker for the prognostic clinical response of Nivolumab treatment in patients with advanced ESCC.^22^ Koyama et al 68previously proved that the failure in PD‐1 blockade was related to the upregulation of Tim‐3. A study investigating the expression of T‐cell immune checkpoints in patients with esophageal cancer showed that the expression of costimulatory molecules was downregulated and the coinhibitory receptor Tim‐3, PD‐1, CTLA‐4, and CD160 was upregulated compared with the normal donors. The expression of PD‐L1 and Tim‐3 on T cells, tumor tissue and PBMC was significantly positively correlated and this relationship was also found in the expression of PD‐L1 and T cell Ig and ITIM domain (TIGIT). The author conceived that these molecules are coexpression patterns, which may result in T‐cell exhaustion.^69^

## 5 MANAGEMENT OF IMMUNOTHERAPY‐RELATED TOXICITIES

The toxic side effects of immunotherapy have always been a stumbling block in the clinical promotion of this treatment and the occurrence of immunological side effects is related to the reduction in immunosuppression. Cutaneous, gastrointestinal, endocrine and hepatic toxicity are the most frequent immune‐related adverse events (irAEs). Because all of these irAEs may occur at any time, careful monitoring, follow‐up, and timely management are required. According to the classification of adverse events, the management methods are different. In general, it includes termination of immunotherapy and initiation of oral or intravenous steroid treatment. Timely and effective treatment can reverse side events. Therefore, we need clinicians to diagnose and take effective measures to manage these side effects.

## 6 CONCLUSION

Establishing a new therapeutic paradigm in esophageal cancer is highly anticipated. Multi‐modal combination therapy is a hot topic discussed by experts, including immunotherapy combined with surgery, chemotherapy, radiotherapy, targeted therapy, and immunotherapy. Existing evidence indicates that the abscopal effect of radiotherapy is based on the immune function of the body. Previous clinical trials have reported that the abscopal effect occurs in the combination of anti‐PDL1 or anti‐PD1 and radiotherapy. Concurrent radiotherapy plus immune checkpoint inhibitor is also superior to sequential therapy.^70^ These provide a basis for immunotherapy combined with traditional treatment. Multiple studies have shown that the expression rate of biomarkers can be increased after chemotherapy, but whether the increase of expression rate of biomarkers can improve the efficiency of immunotherapy is still worth exploring. Because of the severe side effects of combination therapy, immunotherapy needs to be fully evaluated. Although there are many biomarkers that can predict the prognosis of immunotherapy, the effective rate is still only 30%. We need to find more effective biomarkers to fully evaluate the immune status of patients and predict the effectiveness of immunotherapy before treatment. Similarly, we also need to explore the causes of treatment failure, and perhaps new treatment directions will be discovered in the process. However, the methods of biomarker evaluation and the cut‐offs for positivity are not unified in different clinical trials, so the reliability of all experimental conclusions remains to be considered. In addition, at this stage, immunotherapy is only a salvage treatment for advanced patients with esophageal cancer. We expect more clinical trials to confirm whether immunotherapy can achieve better results in early applications. Because the mechanism of immunotherapy is different from other treatments and the increased tumor burden or appearance of new lesions in the short term after immunotherapy have been confirmed. Therefore, it is worth suspecting whether the traditional evaluation principle is still applicable in immunotherapy.

## CONFLICT OF INTERESTS

The authors declare that they have no competing interests.

## AUTHORS’ CONTRIBUTION

QZ designed the study and drafted the manuscript. JMY and XM coordinated, edited, and finalized the drafting of manuscript. All authors read and approved the final manuscript.

## Data Availability

The dataset supporting the conclusions of this article is included within the article.
